# The role of microglial inflammasome activation in pyroptotic cell death following penetrating traumatic brain injury

**DOI:** 10.1186/s12974-019-1423-6

**Published:** 2019-02-08

**Authors:** Stephanie W. Lee, Juan Pablo de Rivero Vaccari, Jessie S. Truettner, W. Dalton Dietrich, Robert W. Keane

**Affiliations:** 1 Department of Neurological Surgery, The Miami Project to Cure Paralysis, University of Miami Miller School of Medicine, Miami, Florida USA; 2 Department of Physiology and Biophysics, University of Miami Miller School of Medicine, Miami, Florida USA

**Keywords:** Inflammasome, ASC, Pyroptosis, Microglia, Infiltrating leukocytes, Traumatic brain injury, Penetrating traumatic brain injury, PTBI, Penetrating ballistic-brain injury, PBBI

## Abstract

**Background:**

Traumatic brain injury remains a significant cause of death and disability in the USA. Currently, there are no effective therapies to mitigate disability except for surgical interventions necessitating a need for continued research into uncovering novel therapeutic targets. In a recent study, we used a rodent model of penetrating traumatic brain injury known as penetrating ballistic-like brain injury (PBBI) to examine the role of innate immunity in post-traumatic secondary injury mechanisms. We previously reported that the inflammasome, a multiprotein complex composed of apoptosis-associated speck-like protein containing card and caspase-1, plays a role in secondary cell death mechanisms after PBBI, including inflammatory cell death (pyroptosis).

**Methods:**

In the current study, we used flow cytometry analysis to evaluate activated microglia and CD11b-positive leukocytes after PBBI and assessed inflammasome activation and pyroptosis of specific cellular populations. Sprague-Dawley male rats underwent PBBI or sham-operated procedures and ipsilateral cortical regions processed for flow cytometry and cellular analysis. Flow cytometry results were compared using one-way ANOVA followed by Tukey’s multiple comparisons.

**Results:**

At 48 h following PBBI, there was an increase in activated microglia and infiltrating leukocytes compared to sham controls that were associated with increased caspase-1 activity. Using a florescent probe to identify caspase-1 activity and a fluorescent assay to determine cell viability, evidence for pyroptosis in CD11b+ cells was also determined. Finally, while post-traumatic treatment with an anti-ASC antibody had no effect on the number of activated microglia and infiltrating leukocytes, antibody treatment decreased caspase-1 activity in both resident microglia and infiltrating leukocytes and reduced pyroptotic CD11b+ cell death.

**Conclusions:**

These results provide evidence for inflammasome activation in microglia and infiltrating leukocytes after penetrating traumatic brain injury and a role for pyroptotic cell death in the pathophysiology. In addition to inhibiting neuronal cell death, therapeutic treatments targeting inflammasome activation may also provide beneficial effects by reducing the potentially detrimental consequences of activated microglia and infiltrating CD11b+ leukocytes following penetrating traumatic brain injury.

## Background

Penetrating traumatic brain injury (PTBI) includes all traumatic brain injuries that are not the result of a blunt mechanism [1–3]. PTBI remains one of the most devastating and lethal forms of trauma, and the prognosis is generally poor [1, 4–6]. The underlying cell death mechanisms contributing to tissue loss following PTBI are multifactorial and incompletely understood [7–10]. Several reports have suggested that cells in the injury core undergo a variety of cell death mechanisms, including necrosis and apoptosis [9, 11–15]. Recently, we used a rodent model of penetrating ballistic-like brain injury (PBBI) [8, 14] that recapitulates cranial gunshot wound pathology and reported that progressive tissue loss following PTBI involves inflammasome activation resulting in initiation of the pyroptosis [16], a form of inflammatory cell death [17–21].

As part of the pyroptotic process, damaged neural cells form specks that are composed of apoptosis speck-like staining protein containing a caspase recruitment domain (ASC) and caspase-1 [22, 23]. ASC specks are released from cells undergoing pyroptosis into the extracellular space resulting in cleavage of pro-interleukin-1β (pro-IL-1β) into its active form [24]. ASC specks may be also taken up by phagocytic cells around the injury periphery, including the infiltrating leukocytes and the resident-activated microglia [25]. The precise cell types undergoing pyroptosis after PTBI remain unclear although previous studies have provided evidence that this type of cell death can occur in neurons and inflammatory cell populations under a variety of conditions [20, 26–29].

Microglia play an important role in the immune system of the central nervous system (CNS) by acting as antigen presenting cells and surveying tissue for immunogens [30–34]. Importantly, microglia initiate the innate immune response by binding danger-associated molecular patterns released by dead or dying cells after CNS injury resulting in inflammasome activation [34–36]. Activated microglia produce pro-inflammatory cytokines and undergo a morphological change from resting to ameboid phagocytic microglia [37, 38]. Traditionally, microglia activation has been categorized into two subtypes, M1 and M2 [39–41]. The M1 phenotype is pro-inflammatory, potentiating neuronal injury, whereas the M2 phenotype is anti-inflammatory, pro-regenerative, and phagocytic [40, 41]. Most recently, additional research including transcriptomic analysis of the M1 and M2 phenotypes has been revealed that microglial polarization is multidimensional rather than linear and that the different microglial activation states have considerable overlap of gene expression [42–45]. The detailed information regarding microglial phenotypes and their contribution to cell death processes following TBI is lacking.

In the present study, we sought to extend our previous investigations using the PBBI model [16] to clarify the role of microglial activation and immune cell infiltration in cell death processes following brain injury. For this goal, we used quantitative flow cytometry analysis to determine the cell-type expression pattern of inflammasome proteins and cells undergoing pyroptosis following PBBI. We report significant increases in inflammasome protein expression in microglia and infiltrating leukocytes in the cerebral cortex, which leads to the pyroptotic cell death. In addition, treatment of traumatized animals with a neutralizing antibody to the inflammasome component, ASC (anti-ASC), significantly decreased caspase-1 activity and pyroptosis in microglia and infiltrating leukocytes. These studies illustrate that microglia and infiltrating immune cells actively participate in the innate immune response after PBBI and undergo pyroptosis contributing to cell loss after injury.

## Methods

### Animals

Animal procedures were approved by the University of Miami’s Institution of Animal Care and Use Committee and adhered to the ARRIVE guidelines and those established by the National Institute of Health Guide for the Care and Use of Laboratory Animals. Male Sprague-Dawley rats (280–350 g) aged 3–5 months were used for all experiments. Male rats were used in this initial study because males are at higher risk of traumatic brain injury (TBI) than females, with the highest male-to-female ratio occurring in adolescence and young adulthood [46]. However, future studies with female rats will have to be conducted to assess potential sex-dependent effects on the inflammatory response after PBBI. To increase scientific rigor, rats were randomly assigned to each experimental group (sham, PBBI + PBS, PBBI + anti-ASC) and a power analysis was carried out to determine sample size calculation based on prior biochemical studies in the PBBI model [47–50].

### PBBI surgery

Rats underwent PBBI or sham procedures as previously described [16]. Rats were anesthetized with 2–5% isoflurane delivered in a mixture of 70% nitrous oxide and 30% oxygen and body temperature maintained at normothermia (37 ± 1 °C) by a homoeothermic heating pad. The rat was secured in the stereotaxic device for insertion of the PBBI probe. The penetrating probe (Kadence Science, Lake Success, NY) which is made of a 20-gauge stainless steel tube with fixed perforations along one end which are sealed by a piece of airtight elastic tubing was secured on the probe holder. A burr hole (diameter 4 mm) over the right frontal pole at 4.5 mm anterior-posterior and + 2 mm medial-lateral to bregma was created using a dental drill as previously described [51]. The PBBI probe was next advanced into the right hemisphere to a depth of 12 mm from the surface of the brain. The pulse generator was activated to release a pressure pulse calibrated to produce a rapid expansion of the water-filled elastic tubing to induce an elliptical-shaped balloon (diameter = 0.633 mm, duration = 40 ms) to a volume equal to 10% of the total brain volume. As previously described, this rapid inflation/deflation mimics the generation of a ballistic force shock wave, thereby creating a temporary unilateral cavity in the brain [8]. After deflation, the probe was removed and the skin incision closed with wound clips. Sham animals received vehicle (PBS, intravenous [i.v.]) injection through the jugular vein 4 h after sham surgery. PBBI animals received either vehicle or anti-ASC (5 mg/kg i.v.) injection through the jugular vein 4 h after PBBI surgery. This dose of anti-ASC was used because it has previously been reported to reduce inflammasome activation in another model of TBI [52]. In this study, we tested PBS as a vehicle since in a previous TBI study, significant reductions in the processing of caspase-1, IL-1B, deceased XIAP cleavage and reduced contusion volume with anti-ASC treatment compared to IgG control of the same isotype [53]. Also, recent studies have indicated that immunoglobulin may actually affect inflammasome activation in some experimental settings so IgG does not appear to be a proper control for assessing inflammasome signaling. Sham surgeries consisted of the midline scalp incision, the right frontal burr hole, without probe insertion. Following surgery, animals were monitored to ensure they did not develop postoperative infections or experience discomfort.

### Flow cytometry

At 48 h post-surgery, all rats were anesthetized with a high dose of 2–5% isoflurane for 5 min and perfused transcardially with ice-cold PBS for 6 min. The ipsilateral cerebral cortex was dissected on ice and placed in ice-cold Hank’s Balanced Salt Solution. The methods used for flow cytometry have been recently described for cerebral cortical and hippocampal analysis [54, 55]. Briefly, cortical brain tissue was mechanically dissociated into a single-cell suspension by passage through a 40-μm cell strainer (Falcon, Madison, WI) and lysed with ACK buffer (Life Technologies, Grand Island, NY). Cells were labeled for caspase-1 activity using a FAM-FLICA assay (Immunochemistry Technologies, Bloomington, MN) following the manufacturer’s instructions. The FLICA Caspase-1 Reagent (FAM-YVAD-FMK) forms an irreversible covalent bond with the fluoromethyl ketone (FMK) to the cleaved active form of caspase-1 (Immunochemistry Technologies). The carboxyfluorescein (FAM) optimally excites at 488–492 nm and has a peak emission at 515–535 nm. The LIVE/DEAD Fixable Near-IR Dead Cell Stain was used for cell viability (L10119, 1 μL/mL, Life Technologies). Following a non-specific block with cluster of differentiation (CD)16/CD32 antibody, cells were labeled for surface markers CD45 Alexa 647 (202212, 1.25 μg/mL, BioLegend) and CD11b v450 (53-4321-80, 1 μg/mL, eBioscience). CD45 and CD11b were used to distinguish between CD45_low_ “resting” microglia (CD45_low_, CD11b+), CD45_intermediate_-activated microglia (CD45_int_, CD11b+), and CD45_high_-infiltrating myeloid-lineage cells (CD45_high_, CD11b+). Activated microglia increase their expression of CD45 compared to steady state surveying microglia while infiltrating leukocytes, including macrophages, monocytes, and neutrophils, express the highest amounts of CD45 [56, 57]. Cells were then fixed with BD Cytofix/Cytoperm Fixation/Permeabilization Kit (554714, BD Biosciences). Gates were established using antibody isotype controls (provided by manufacturers) and fluorescence minus one controls. The samples were acquired on Beckman Coulter CytoFLEX S using CytExpert 2.0 as acquisition software. The resulting FCS files were analyzed with Kaluza 1.5A (Beckman Coulter) software.

### Scientific rigor and statistical analysis

Rats were randomly assigned to each experimental group including sham operated, PBBI + PBS, and PBBI + anti-ASC (*n* = 5 per group). Samples were coded prior to flow cytometry, and the codes were broken after analysis with Kaluza and prior to statistical analysis. Statistical analyses were performed using Prism 6 (GraphPad Software, Inc., La Jolla, CA, USA). All measures were expressed as mean ± standard error of the mean with *p* ≤ 0.05 considered significant in all statistical tests. Flow cytometry results were compared using one-way ANOVA followed by Tukey’s multiple comparisons.

## Results

### PBBI induces an increase in activated microglia and infiltrating leukocytes, and anti-ASC treatment has no significant effect on numbers of activated microglia or infiltrating leukocytes

**Fig. 1:**
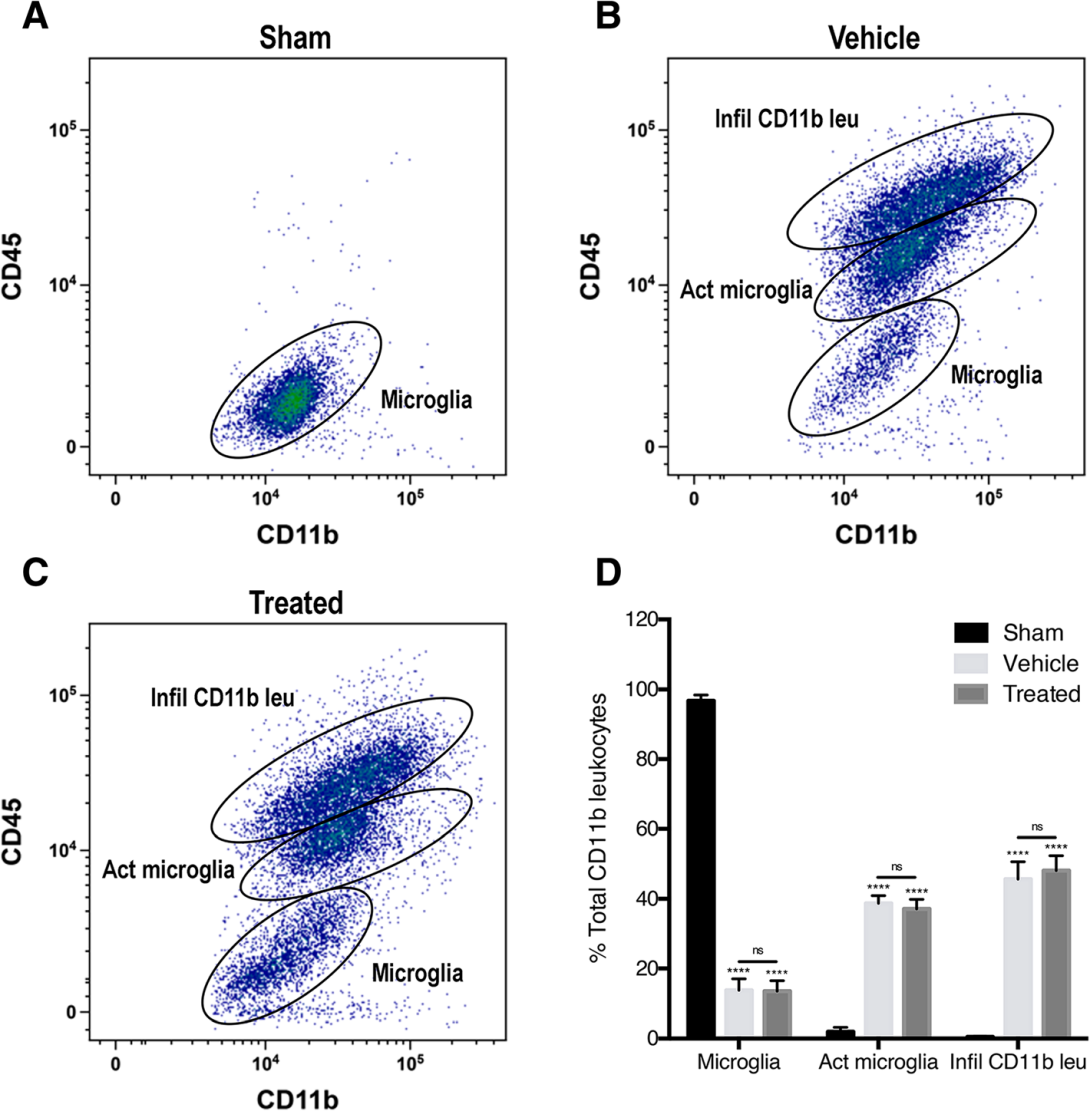
Representative flow cytometry density plots from the ipsilateral cortex of **a** sham, **b** PBBI injured (vehicle), and **c** PBBI treated with anti-ASC. **d** Quantification of the number of surveying microglia, activated microglia, and infiltrating CD11b+ leukocytes at 48 h post-surgery. PBBI significantly increased the number of activated microglia and infiltrating CD11b+ leukocytes 48 h after injury. There was no change in either the number of infiltrating CD11b+ leukocytes or the number of microglia after treatment with anti-ASC. Data are presented as mean ± standard error of the mean. Statistical significance was determined with one-way ANOVA followed by Tukey’s post hoc test. ****p < 0.0001. ns, no significance. *n* = 6 per group

Flow cytometry analysis of cortical tissue was used to separate populations of resident microglia and infiltrating cells including neutrophils and macrophages as previously described [55]. Cells were labeled with CD11b which is a surface marker for neutrophils, macrophages, and microglia. Additionally, cortical cells are labeled with CD45 which is a cell surface marker for leukocytes. The cells that were positive for CD11b but not for CD45 indicated included resident microglia, and those that were positive for both CD11b and CD45 indicated infiltrating neutrophils and monocytes, including macrophages. Forty-eight hours after PBBI, density plots showed increased markers of microglial and infiltrating leukocytes in all animals compared to sham-operated animals. Microglia in sham cortices expressed a low amount CD45 (CD45_low_, CD11b+), as evidenced as tight cell clusters on the graph with very few cells in the CD45_int_ and CD45_high_ range (Fig. 1).

Following PBBI, cells in vehicle and anti-ASC treated cortices were gated into three populations: surveying microglia (CD45_low_, CD11b+), activated microglia (CD45_int_, CD11b+), and infiltrating CD11b+ leukocytes (CD45_high_, CD11b+). Although, there were significantly less surveying microglia in the vehicle and treated animals 48 h after PBBI compared to sham animals. However, there was no significant difference between vehicle and treated groups. The CD45 and CD11b expression pattern of surveying microglia (CD45_low_, CD11b+) in vehicle and treated cortices differed from the expression pattern of surveying microglia (CD45_low_, CD11b+) in sham cortices showing that the cells were less tightly clustered indicating that the microglia increased their expression of both CD45 and CD11b after injury when compared to sham. Conversely, there were significantly more activated microglia (CD45_int_, CD11b+) in vehicle and treated animals 48 h post-PBBI compared to sham animals. Treatment with anti-ASC did not significantly change the number of activated microglia when compared to vehicle. Compared to sham, the number of infiltrating CD11b+ leukocytes (CD45_high_, CD11b+) also significantly increased in vehicle and treated animals 48 h after injury. Similar to the number of activated microglia (CD45_int_, CD11b+), the number of infiltrating leukocytes (CD45_high_, CD11b+) did not significantly change after treatment with an inhibitor of ASC. These results indicate that PBBI increases the number of activated microglia (CD45_int_, CD11b+), recapitulating stereology results from a previous study [16], and the number of infiltrating leukocytes (CD45_high_, CD11b+). However, anti-ASC treatment did not alter the number of either type of cell.

### Caspase-1 activity increases in activated microglia and infiltrating leukocytes 48 h after PBBI and decreases after treatment with anti-ASC

**Fig. 2:**
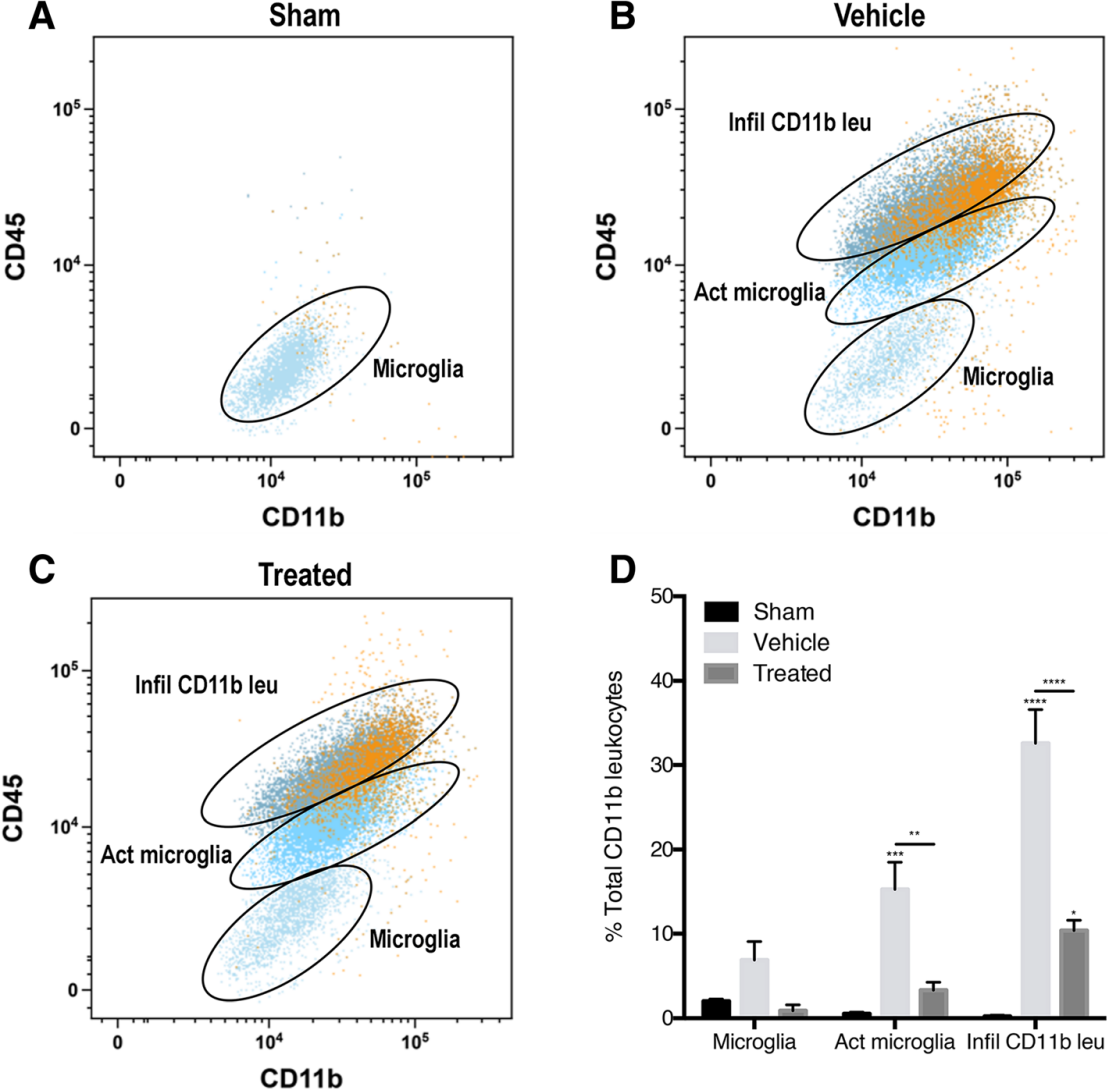
Representative flow cytometry scatter plots of infiltrating CD11b+ leukocytes and microglia expressing caspase-1 activity from the ipsilateral cortex of **a** sham, **b** PBBI injured (vehicle), and **c** PBBI treated with anti-ASC. **d** Quantification of the number of “resting” microglia, activated microglia, and infiltrating CD11b+ leukocytes 48 h after PBBI. Forty-eight hours after injury, PBBI significantly increased the number of activated microglia and infiltrating CD11b+ leukocytes expressing caspase-1 activity. Treatment with an antibody inhibiting ASC significantly decreased the amount of caspase-1 activity in both activated microglia and infiltrating CD11b+ leukocytes. Data are presented as mean ± standard error of the mean. Statistical significance was determined with one-way ANOVA followed by Tukey’s post hoc test. *p < 0.05, **p < 0.01, ***p < 0.001, ****p < 0.0001. *n* = 5 per group

To determine the role of ASC on caspase-1 activity in microglia and infiltrating leukocytes in PBBI, PBBI animals were treated with anti-ASC, a biological inhibitor of adaptor protein ASC, and flow cytometry was conducted to measure caspase-1 activity before and after treatment. A FAM-FLICA florescent probe, which only binds to cleaved and activated caspase-1, was used to measure caspase-1 activity in cells isolated from the cortices. Using the established gates for CD45 and CD11b, caspase-1 activity significantly increased in the activated microglia (CD45_int_, CD11b+) and infiltrating CD11b+ leukocytes (CD45_high_, CD11b+) in vehicle animals 48 h after PBBI (Fig. 2). While there was a moderate increase in caspase-1 activity in surveying microglia, the increase was not significant. Inhibition of ASC, in the treated animals, resulted in a decrease in caspase-1 activity in the activated microglia population (CD45_int_, CD11b+) and the infiltrating leukocytes population (CD45_high_, CD11b+). These results suggest that PBBI increases caspase-1 activity in activated microglia and infiltrating CD11b+ leukocytes and that inhibition of ASC decreases caspase-1 activity in these cells. Therefore, inflammasome protein ASC contributes to inflammasome activation and caspase-1 activity in microglia and infiltrating leukocytes in PBBI.

### PBBI increases caspase-1 activity and pyroptosis in CD11b+ cells after PBBI, and anti-ASC decreases the number of CD11b+ cells undergoing pyroptosis

**Fig. 3:**
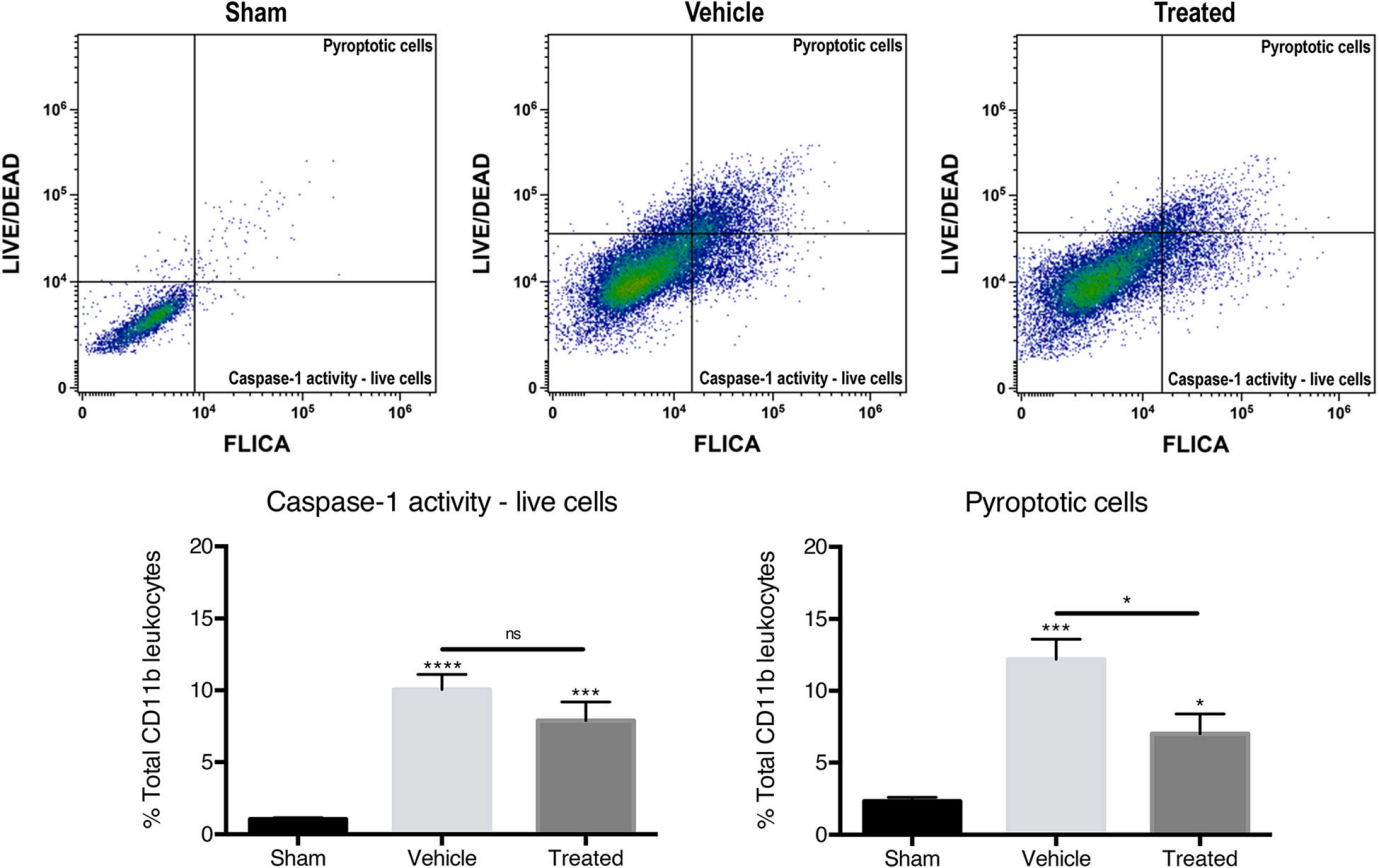
Representative flow cytometry density plots of caspase-1 activity (FLICA) versus amine reactivity (LIVE/DEAD) from the ipsilateral cortex of sham, PBBI injured (vehicle), and PBBI treated with anti-ASC (top row). CD11b+ cells were gated for pyroptotic cells (high FLICA expression and high LIVE/DEAD expression) and caspase-1 activity live cells (high FLICA expression and low LIVE/DEAD expression). Quantification of the number of CD11b+ cells that are live cells expressing caspase-1 activity or cells undergoing pyroptosis 48 h post-injury (bottom row). PBBI significantly increased the number of CD11b+ live cells expressing caspase-1 activity and the number of CD11b+ cells undergoing pyroptosis. The number of CD11b+ cells undergoing pyroptosis significantly decreased after treatment with anti-ASC. Data are presented as mean ± standard error of the mean. Statistical significance was determined with one-way ANOVA followed by Tukey’s post hoc test. *p < 0.05, **p < 0.01, ***p < 0.001, ****p < 0.0001. *n* = 5 per group

To determine the role of ASC in pyroptosis and caspase-1 activity in CD11b+ cells after PBBI, animals were treated with anti-ASC and flow cytometry was conducted to measure cell viability and caspase-1 activity. In addition to the FAM-FLICA florescent probe, a florescent probe against amine residues of proteins (LIVE/DEAD) was used to determine cell viability [58]. The LIVE/DEAD assay is membrane impermeable and binds to amines of membrane proteins when the cell membrane is intact resulting in low levels of florescence. Dead or dying cells become porous membrane and allow the LIVE/DEAD to permeate the cell, thereby increasing the florescence of the cells. Plotting the CD11b+ cells using FLICA florescence and LIVE/DEAD florescence establishes four quadrants of gates: live CD11b+ cells that do not express caspase-1 activity (FLICA_low_, LIVE/DEAD_low_), live CD11b+ cells that express caspase-1 activity (FLICA_high_, LIVE/DEAD_low_), necrotic CD11b+ cells (FLICA_low_, LIVE/DEAD_high_), and pyroptotic CD11b+ cells (FLICA_high_, LIVE/DEAD_high_) (Fig. 3 top row). Using these gates, caspase-1 activity in live cells and pyroptosis was found to be increased in all CD11b+ cortical cells 48 h after PBBI (Fig. 3 bottom row). There was no significant difference in caspase-1 activity in live CD11b+ cells after inhibition of ASC, but treatment with anti-ASC significantly decreased pyroptosis in CD11b+ cells. These results indicate that PBBI increases cell death by pyroptosis in the cortex, supporting the immunoblot results of pyroptosome formation and gasdermin-D (GSDMD) expression seen in previous studies [16] (Fig. 3), and that inhibition of ASC decreases pyroptotic cell death. Therefore, inflammasome adaptor protein ASC contributes to the pyroptosis of CD11b+ cells.

### PBBI increases caspase-1 activity and pyroptosis in infiltrating leukocytes and activated microglia after PBBT, and anti-ASC treatment decreases the number of infiltrating leukocytes that undergo pyroptosis

**Fig. 4:**
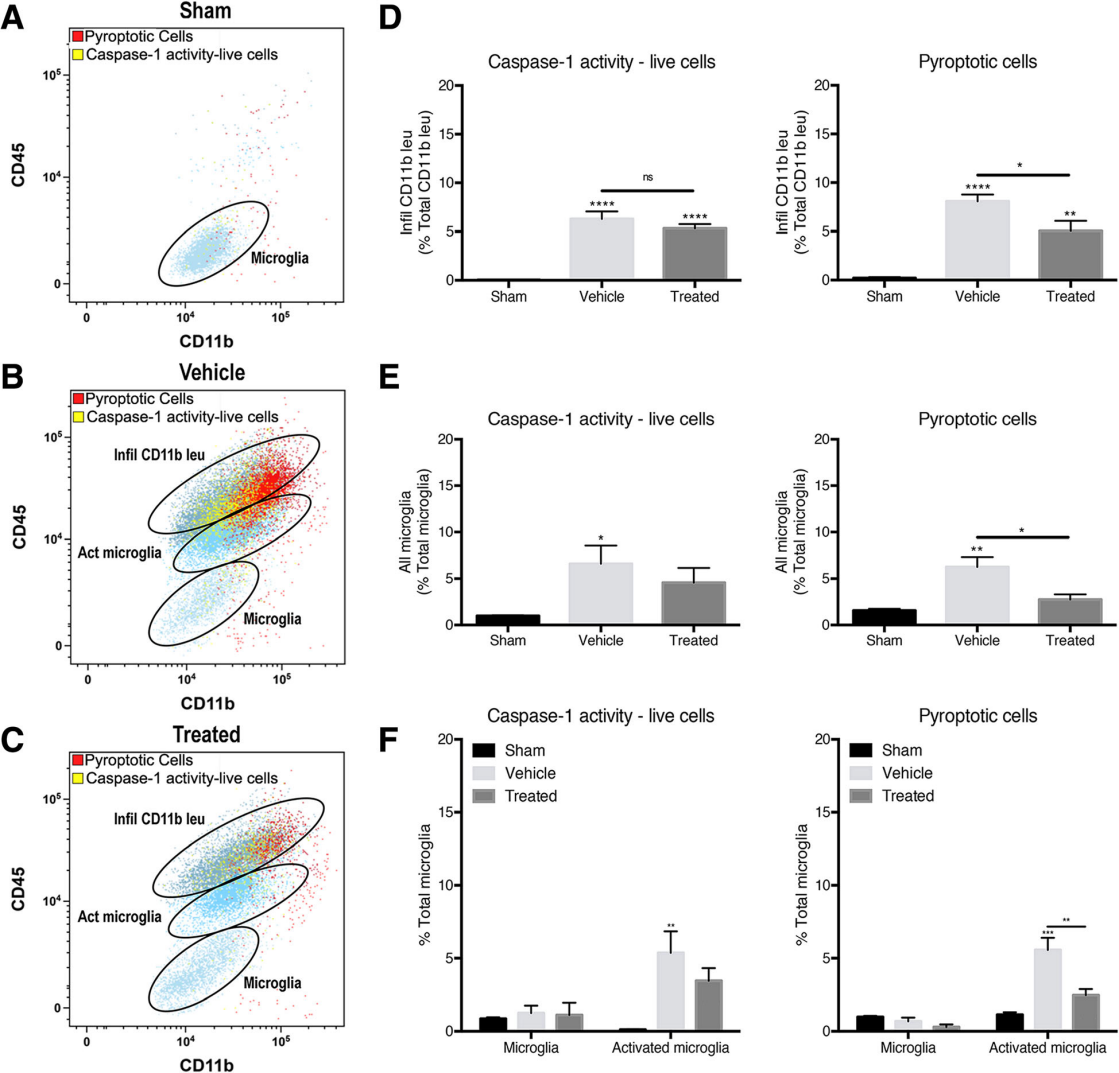
Representative flow cytometry scatter plots of infiltrating CD11b+ leukocytes and microglia that are pyroptotic or caspase-1 activity expressing live cells from the ipsilateral cortex of **a** sham, **b** PBBI injured (vehicle), and **c** PBBI treated with anti-ASC. Cells previously gated for pyroptosis or live cells expressing caspase-1 activity were further gated into the three populations of cells: infiltrating CD11b+ leukocytes, activated microglia, and microglia. **d** Quantification of the number of infiltrating CD11b+ leukocytes that are live cells expressing caspase-1 activity and cells undergoing pyroptosis (expressing both increased amine reactivity and caspase-1 activity) 48 h post-injury. PBBI significantly increased the number of pyroptotic and caspase-1 activity-live infiltrating CD11b+ leukocytes. Treatment with an antibody against ASC significantly decreased the amount of infiltrating CD11b+ leukocytes undergoing pyroptosis. **e** Quantification of “all” microglia (surveying microglia + activated microglia). **f** Surveying microglia versus activated microglia that are live cells expressing caspase-1 activity or pyroptotic cells 48 h post-surgery. The number of pyroptotic microglia and live microglia expressing caspase-1 significantly increased after PBBI while treatment inhibiting ASC significantly decreased microglia undergoing pyroptosis. Splitting of the microglia population into surveying phenotype versus activated phenotype showed that activated microglia were the significant contributors to the number of live microglia expressing casepase-1 and overall microglial pyroptosis after PBBI. Treatment with anti-ASC significantly decreased the number of activated microglia undergoing pyroptosis. Data are presented as mean ± standard error of the mean. Statistical significance was determined with one-way ANOVA followed by Tukey’s post hoc test. *p < 0.05, **p < 0.01, ***p < 0.001, ****p < 0.0001. *n* = 5 per group

To determine the role of ASC in pyroptosis and caspase-1 activity in microglia and infiltrating CD11b+ leukocytes in PBBI, animals were treated with an inhibitor of ASC and flow cytometry was conducted to determine cell viability and caspase-1 activity. Using the pyroptotic cell gate (FLICA_high_, LIVE/DEAD_high_) and caspase-1 activity live cell (FLICA_high_, LIVE/DEAD_low_) gate, the CD11b+ cells were further gated into “resting” microglia (CD45_low_, CD11b+), activated microglia (CD45_int_, CD11b+), and infiltrating leukocytes (CD45_high_, CD11b+) (Fig. 4 a–c). Sham brains show very few pyroptotic cells (red) and caspase-1 active live cells (Fig. 4 a). After PBBI, in both vehicle and treated cortices, the pyroptotic cells and live cells with caspase-1 activity were predominant in the infiltrating CD11b+ leukocyte gate (CD45_high_, CD11b+) and activated microglia gate (CD45_int_, CD11b+) (Fig. 4 b, c). In the infiltrating CD11b+ leukocyte population, there was a significant increase in the number of live cells with caspase-1 activity and cells undergoing pyroptosis in the injured cortices 48 h after PBBI (Fig. 4 d). After treatment with anti-ASC, there was a significant decrease in the number of pyroptotic cells; however, there was no significant change in the number of caspase-1 active live cells. In all microglia (“resting” microglia + activated microglia), there was a significant increase in pyroptotic cells and caspase-1-activated live cells and treatment with an ASC inhibitor decreased pyroptosis (Fig. 4 e). Dividing the microglia into the “resting” population (CD45_low_, CD11b+) versus activated population (CD45_int_, CD11b+), it was observed that activated microglia were the main source of the live cells with caspase-1 activity and pyroptotic cells in PBBI cortices (Fig. 4 f). Treatment with an inhibitor of ASC significantly decreased the number of microglia undergoing pyroptosis, specifically decreasing the number of activated microglia undergoing pyroptosis (Fig. 4 e, f). These results suggest that activated microglia and infiltrating CD11b+ leukocytes express caspase-1 activity and undergo pyroptosis 48 h after PBBI and that treatment with anti-ASC lessens the extent of pyroptotic cell death. Therefore, inflammasome protein ASC contributes to inflammasome activation and pyroptosis of activated microglia and infiltrating CD11b+ leukocytes after PBBI.

## Discussion

This study provides several new findings that clarify the underlying pathophysiology of secondary injury mechanisms after severe TBI. We present evidence using flow cytometry density plots that PBBI significantly increases the number of activated microglia and infiltrating CD11+ leukocytes 48 hrs after injury. Likewise, the number of activated microglia and infiltrating CD11+ leukocytes expressing caspase-1 was also significantly increased in cortical inflammatory cells compared to sham-operated animals. Using a FAM-FLICA florescent probe and LIVE/DEAD assay, caspase-1 activity in live and pyroptotic cells was found to be increased in all CD11+ cortical cells after PBBT. Post-traumatic treatment with an antibody inhibiting ASC significantly decreased the amount of caspase-1 activity in both activated microglia and CD11+ leukocytes and reduced the number of CD11+ leukocytes undergoing pyroptosis. Taken together, these new findings indicate that activation of the inflammasome and pyroptosis contributes to the early pro-inflammatory response to severe TBI and supports previous findings showing pyroptosome formation and gasdermin-D expression after PBBT [16]. In addition, it is suggested that therapeutic interventions targeting abnormal inflammasome activation after severe TBI represent a novel approach to limiting secondary injury mechanisms by targeting both neuronal and inflammatory cell death.

Although previous studies have reported NLRP3 inflammasome activation in microglia and macrophage populations under several experimental conditions [16, 38, 59–62], few have demonstrated the specific mechanism of cell death induced by inflammasome activation. In a recent study, Lee and colleagues reported following PTBI that neurons and microglia appeared to be the predominant cell types expressing inflammasome proteins between 24 and 48 h after injury using immunocytochemistry [16]. In terms of inflammasome-mediated cell death, Kim and colleagues demonstrated that *Streptococcus pneumoniae* infection induced pyroptosis in cultured microglia as evidenced by cleavage of caspase-1 and an increase in lactate dehydrogenase release into the culture media [27]. Pyroptosis is a caspase-1-dependent process that results in programmed cell death [63, 64], and there is limited information on measures of microglial and macrophage pyroptosis using in vivo models of TBI. Here, we used flow cytometry to demonstrate significant caspase-1 activation and cell death in activated microglia and infiltrating leukocytes using an established model of PTBI. We assessed pyroptosis by measuring various parameters within the same cell including caspase-1 activity via the YVAD domain of the FLICA assay and cell viability via a LIVE/DEAD assay. Together, these findings indicate that pyroptosis of activated microglia and infiltrating leukocytes may act to amplify the pro-inflammatory response to PBBI injury that may participate in the structural and functional abnormalities seen in this penetrating brain injury model [8, 9, 16, 65, 66].

Our flow cytometry experiments revealed an increase in the number of activated microglia 48 h after PBBI that corresponds to increased microglia previously assessed by stereological counts using the same model [16]. These findings are also in agreement with published data using other TBI models reporting increases in microglia numbers and in human TBI in post-mortem brain sections [33, 40, 44, 55, 67]. The increase in infiltrating CD11b+ leukocytes, including macrophages and neutrophils, after PBBI is also consistent with previous reports of increased inflammatory cell infiltration and associated alterations in vascular permeability [47, 49]. In this study, we used CD11d and CD45 for the flow cytometry analysis to differentiate endogenous microglia from infiltrating leukocytes. While ramified parenchymal microglia possess the phenotype CD11b^+^/CD45^low^, other CNS macrophages and peripheral macrophages exhibit the phenotype CD11^+^/CD45^high^. Thus, while both CD11b and CD45 can recognize various subtypes of invading cells including leukocytes and lymphocytes that may participate in the pathophysiology of TBI, the current strategy allowed us to isolate these two major inflammatory cell populations with flow cytometry to evaluate inflammasome signaling.

To reduce the detrimental consequences of pro-inflammatory processes after TBI, various therapeutic targets and strategies have been investigated with mixed results [54, 68–70]. The anti-inflammatory and neuroprotective drug NNZ-2566 has been reported to be neuroprotective in PBBI [49]. In that study, NNZ-2566 treatment increased both mRNA and protein levels of activating transcription factor-3 in multiple cell types following PBBI and decreased the number of neutrophils and macrophages [49]. In the current study, we investigated the effects of an anti-ASC antibody that has previously been reported to reduce abnormal inflammasome activation in models of brain and spinal cord injury [52, 53, 71, 72]. Importantly, this experimental treatment approach targeting abnormal inflammasome activation after brain and spinal cord injury has also been reported to improve behavioral outcomes and reduce structural damage [53, 71, 72]. Although we did not assess behavioral or histopathological outcomes, we report that anti-ASC treatment decreased the amount of caspase-1 activity in both types of inflammatory cells after PTBI while not decreasing the number of activated microglia or infiltrating CD11b+ leukocytes. Since caspase-1 activity regulates IL-1β processing, the decrease in caspase-1 activity suggests that anti-ASC treatment may block the initiation of the innate immune response leading to pyroptosis [33, 40, 41]. In addition to pro-IL-1β cleavage, caspase-1 also cleaves GSDMD, a protein implicated in pyroptotic pore formation and a necessary step in the pathogenesis of pyroptotic cell death [73]. This plasma membrane pore formation leads to the secretion of IL-1β and subsequent cell lysis [73–75]. Using live-cell imaging of pyroptotic cell death in HEK-293T cells and murine bone marrow-derived macrophages, Rathkey and colleagues [76] have recently clarified the molecular parameters by which GSDMD becomes cleaved and inserts into cellular membranes. The exact mechanisms by which anti-ASC antibody treatment decreases pyroptosis of microglia and infiltrating leukocytes have not been clarified, and additional studies are required to better understand subcellular targets and consequences on brain recovery and function after TBI. For example, strategies including a gene silencing approach targeting NLRP1, NLRP3, or GSDMD could be used to determine how changes in these proteins affect the formation of the inflammasome in microglia or patterns of cellular vulnerability.

**Fig. 5:**
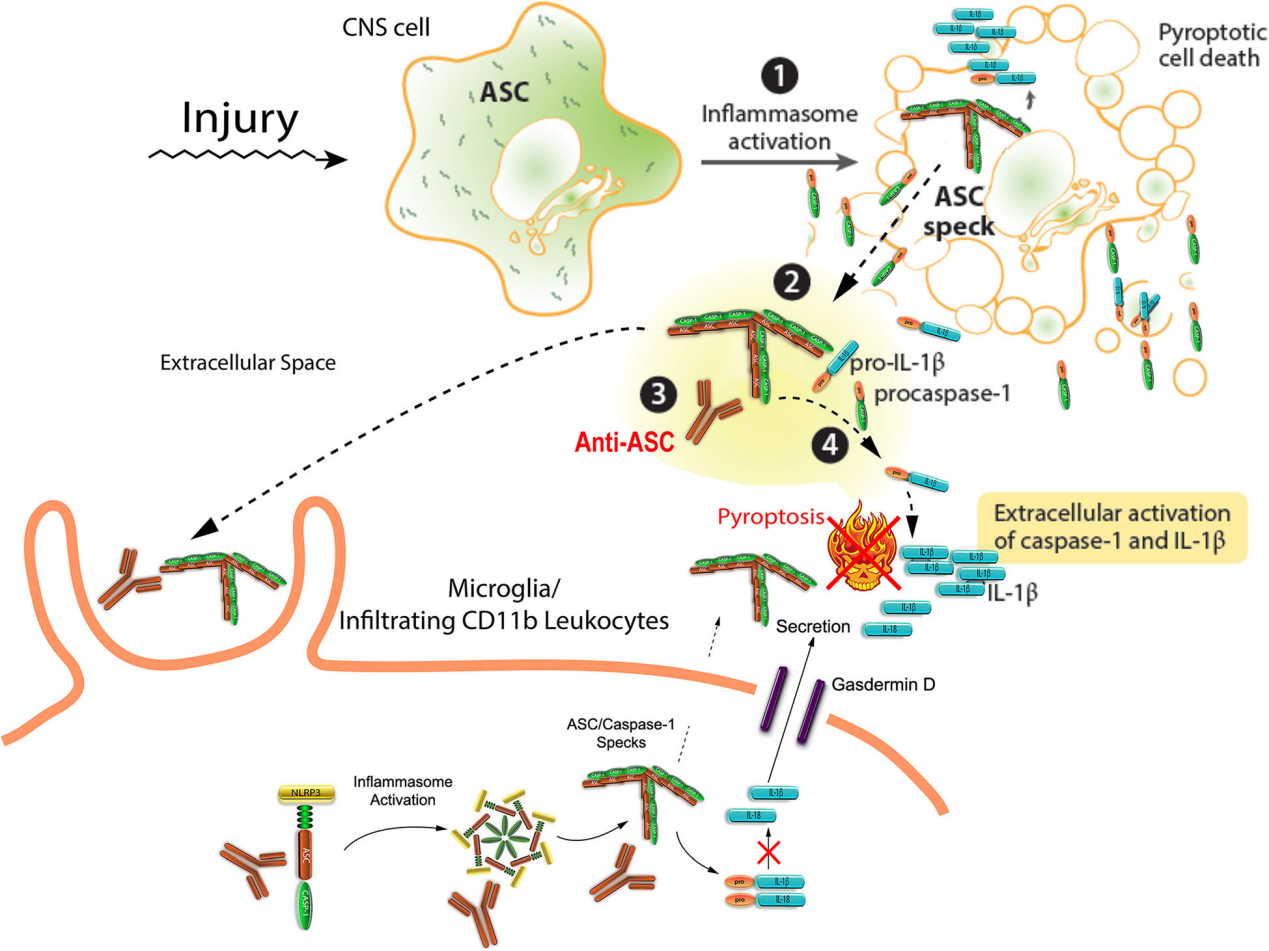
Schematic of inflammasome activation and pyroptosis of microglia after PBBI and proposed effects of anti-ASC on the pathway. CNS injury induces the formation of ASC specks in traumatized cells that are released into the extracellular space leading to maturation of IL-1β. ASC specks are taken up by endogenous microglia or infiltrating phagocytic cells resulting in further inflammasome activation and subsequent death by pyroptosis. Anti-ASC either binds to extracellular ASC specks blocking extracellular IL-1 maturation thereby decreasing inflammasome activation and pyroptosis of microglia and infiltrating CD11b leukocytes or binds to intracellular inflammasomes thereby leading to decreased activation and pyroptosis of microglia and infiltrating CD11b leukocytes. Adapted from Broderick et al. [25]

Based on the current findings and previous studies, it may be hypothesized that in this severe TBI model, inflammatory cell pyroptosis may induce the formation of ASC specks in traumatized cells that are released into the extracellular space leading to maturation of IL-1β [22, 23]. ASC specks are known to be taken up by endogenous microglia or infiltrating phagocytic cells resulting in further inflammasome activation and subsequent death by pyroptosis [25]. The ASC specks have been shown to have “prionoid-like” effects and can also propagate inflammation like IL-1β [23]. Treatment with an anti-ASC antibody may therefore bind directly to extracellular ASC specks blocking extracellular IL-1 maturation and decrease inflammasome activation and pyroptosis of microglia and infiltrating CD11b leukocytes (Fig. 5). Alternatively, the anti-ASC antibody may bind to intracellular inflammasomes, thereby leading to decreased activation and pyroptosis (Fig. 5). The recent discovery that anti-ASC binds to ASC specks and reduces the amount of Aβ monomers and oligomers leading to decreased Aβ deposition [77] suggests that inhibiting ASC may also target progressive pathophysiological mechanisms leading to post-traumatic neurodegenerative disorders including Alzheimer’s disease and dementia.

## Conclusions

In summary, our current findings emphasize that a severe model of PBBI induces significant increases in cell-specific inflammasome protein expression which contributes to the inflammatory cell death of microglia and infiltrating leukocytes. Treatment with an ASC neutralization antibody decreased caspase-1 activity and pyroptosis in microglia and infiltrating leukocytes. These data illustrate that resident and invading inflammatory cells actively participate in the early innate immune response to PBBI. The persistence and mobility of these cells may represent important secondary injury processes that aggravate the injury process and potentially lead to the increased vulnerability of more remote brain regions not initially damaged by the primary injury. Continued investigations into the role of abnormal inflammasome activation in specific cell populations will provide critical information regarding novel cellular and molecular therapeutic targets to reduce the acute and more progressive consequences of TBI.

## Abbreviations

ASC: Apoptosis speck-like staining protein containing a caspase recruitment domain
CD: Cluster of differentiation
CNS: Central nervous system
FAM: Carboxyfluorescein
FMK: Fluoromethyl ketone
IL-1β: Interleukin-1β
PBBI: Penetrating ballistic-like brain injury
PTBI: Penetrating traumatic brain injury
TBI: Traumatic brain injury
